# C4d in IgA nephropathy: a marker sans boundaries

**Published:** 2016-01-09

**Authors:** Muhammed Mubarak

**Affiliations:** Department of Histopathology, Sindh Institute of Urology and Transplantation (SIUT), Karachi, Pakistan

**Keywords:** IgA nephropathy, Oxford classification system, C4d, Rapidly progressive glomerulonephritis

**Implication for health policy/practice/research/medical education:**

IgA nephropathy (IgAN) is the most common glomerulopathy worldwide. After the development of Oxford classification of IgAN, it was established that pathological parameters can also be useful, independent of all clinical or laboratory parameters, in predicting the future course of the disease in individual patients. During the recent past, a novel fragment of the complement activation cascade became the focus of research as a biomarker of current or recent antibody action. This fragment is known as C4d and is now widely used in the investigation of solid organ graft pathology. The current enthusiasm surrounding the role of C4d in the diagnosis and prognosis of both primary and secondary glomerular diseases needs cautionary approach and further investigations are needed to define the exact implications of C4d in clinical practice.

IgA nephropathy (IgAN) is the most common glomerulopathy worldwide (1). In the long-term, the disease is characterized by the development of end-stage renal disease (ESRD) in around 40% of cases at 20-year of follow-up (2,3). The rate of development of this complication varies, and in the majority of cases, the disease slowly progresses to this stage. In a minority of cases, the disease presents as rapidly progressive glomerulonephritis (RPGN) and follows a rapid downhill course to this fate. It is of paramount importance to identify these progressive forms of IgAN and to separate them from the non-progressors. A variety of clinical and laboratory parameters have been used for this purpose and have proved quite useful, especially if done serially and followed over time (4). The use of pathological features on renal biopsies for the purpose of prognostication has been controversial till recent past. One of the limitations of the biopsy-based approach was that the biopsy provided information at one time point in the course of the disease, while the clinical and some laboratory parameters could be repeated multiple times without much discomfort to the patient. However, with the development of Oxford classification of IgAN, it was established that pathological parameters can also be useful, independent of all clinical or laboratory parameters, in predicting the future course of the disease in individual patients (5). This classification has been validated in many studies around the world, in different centers and both in adults and children. However, some of the pathological lesions have not been addressed by this classification in detail and need further investigation (6).

It is well known that the complement system of plasma proteins plays an important role in the pathogenesis and pathology of many renal diseases, especially glomerular diseases (7). A number of complement components are routinely investigated for their deposition in the glomeruli of the kidneys by immunofluorescence, immunohistochemistry or electron microscopy. During the recent past, a novel fragment of the complement activation cascade became the focus of research as a biomarker of current or recent antibody action. This fragment is known as C4d and is now widely used in the investigation of solid organ graft pathology. The Banff classification now recommends routine use of this marker on all dysfunctional renal graft biopsies. More recently, this marker has also been investigated for its potential usefulness in the diagnosis and prognosis of native renal diseases, especially glomerulopathies. Among the later conditions, IgAN is the one in which the role of C4d has been investigated most and the results are promising (8-11). A number of other glomerular diseases have also been investigated using this marker including membranous nephropathy and lupus nephritis. However, the current enthusiasm surrounding the role of C4d in the diagnosis and prognosis of both primary and secondary glomerular diseases needs cautionary approach. We know that mesangial positivity of C4d is a normal occurrence in the glomeruli and serves as positive internal control in renal graft biopsies. Most of the studies on the role of C4d in the glomerular diseases have investigated the deposition of C4d in the mesangium of the glomeruli. It is challenging to distinguish the normal physiological C4d deposition from the abnormal or pathological C4d deposition, especially on immunohistochemistry. The later procedure is technically demanding and poses challenges in the interpretation of results. The results vary from laboratory to laboratory and require strict standardization. Nevertheless, many investigators, including our own group, have investigated the potential role of C4d in a variety of primary and secondary renal diseases of glomerular origin with variable results (8-13).

Rath et al in the current issue of this journal have investigated the role of C4d deposits in the glomeruli and peritubular capillaries of patients with IgAN (14). They investigated the staining intensity and pattern of C4d deposition by immunohistochemistry and correlated it with the MEST parameters of Oxford classification. They concluded that C4d deposition correlated best with the endocapillary proliferation (E) of the Oxford classification. However, there are some limitations in the study, which have also been acknowledged by the authors. These included: small number of cases, with underrepresentation of some of the MEST lesions, use of immunohistochemistry on paraffin-embedded tissue rather than immunofluorescence staining for demonstrating C4d deposits and cross-sectional nature of the study. In spite of the above limitations, the authors were able to draw some useful conclusions from their study and these need to be corroborated further in large-scale and preferably multi-center studies in their country with large number of cases. We hope that the authors will continue their work on this project, preferably in a prospective fashion and expand their study to a multi-center project for further exploring the role of C4d in this disease.

## Author’s contribution

MM was the single author of the manuscript.

## Ethical considerations

Ethical issues (including plagiarism, misconduct, data fabrication, falsification, double publication or submission, redundancy) have been completely observed by author.

## Conflicts of interest

The author declared no competing interests.

## Funding/Support

None.

## References

[1] Mubarak M (2011). IgA nephropathy: an update on pathogenesis and classification. J Coll Physicians Surg Pak.

[2] D’Amico G (1985). The commonest glomerulonephritis in the world: IgA nephropathy. Q J Med.

[3] Geddes CC, Rauta V, Gronhagen-Riska C, Bartosik LP, Jardine AG, Ibels LS (2003). A tricontinental view of IgA nephropathy. Nephrol Dial Transplant.

[4] D’Amico G (2000). Natural history of idiopathic IgA nephropathy: role of clinical and histological prognostic factors. Am J Kidney Dis.

[5] Cattran DC, Coppo R, Cook T, Feehally J, Roberts ISD, Troyanov S (2009). The Oxford classification of IgA nephropathy: rationale, clinicopathologic correlations, and classification. Kidney Int.

[6] Coppo R, Troyanov S, Bellur S, Cattran D, Cook HT, Feehally J (2014). Validation of the Oxford classification of IgA nephropathy in cohorts with different presentations and treatments. Kidney Int.

[7] Kim MK, Maeng YI, Lee SJ, Lee IH, Bae J, Kang YN (2013). Pathogenesis and significance of glomerular C4d deposition in lupus nephritis: activation of classical and lectin pathways. Int J Clin Exp Pathol.

[8] Espinosa M, Ortega R, Gómez-Carrasco JM, López-Rubio F, López-Andreu M, López-Oliva MO (2009). Mesangial C4d deposition: a new prognostic factor in IgA nephropathy. Nephrol Dial Transplant.

[9] Maeng YI, Kim MK, Park JB, Cho CH, Oh HK, Sung WJ (2013). Glomerular and tubular C4d depositions in IgA nephropathy: relations with histopathology and with albuminuria. Int J Clin Exp Pathol.

[10] Espinosa M, Ortega R, Sánchez M, Segarra A, Salcedo MT, González F (2014). Association of C4d Deposition with Clinical Outcomes in IgA Nephropathy. Clin J Am Soc Nephrol.

[11] Val-Bernal JF, Garijo MF, Val D, Rodrigo E, Arias M (2012). C4d as a diagnostic tool in membranous nephropathy. Nefrologia.

[12] Espinosa-Hernández M, Ortega-Salas R, López-Andreu M, Gómez-Carrasco JM, Pérez-Sáez MJ, Pérez-Seoane C (2 14). C4d as a diagnostic tool in membranous nephropathy Nefrologia. 201.

[13] Nasri H, Ahmadi A, Rafieian-kopaei M, Bashardoust B, Nasri P, Mubarak M (2015). Association of glomerular C4d deposition with various demographic data in IgA nephropathy patients; a preliminary study. J Nephropathol.

[14] Rath A, Tewari R, Mendonca S, Badwal S, Nijhawan VS (2016). Oxford classification of IgA nephropathy and C4d deposition; correlation and its implication. J Nephropharmacol.

